# Suicide first aid guidelines for Sri Lanka: a Delphi consensus study

**DOI:** 10.1186/s13033-016-0085-3

**Published:** 2016-08-30

**Authors:** Saranga A. De Silva, Erminia Colucci, Jayan Mendis, Claire M. Kelly, Anthony F. Jorm, Harry Minas

**Affiliations:** 1 Global and Cultural Mental Health Unit, Centre for Mental Health, Melbourne School of Population and Global Health, The University of Melbourne, 207 Bouverie Street, Carlton, VIC 3053 Australia; 2 National Institute of Mental Health, Colombo, Sri Lanka; 3 Mental Health First Aid Australia, Parkville, Australia; 4 School of Psychology, Deakin University, Geelong, Australia; 5 Population Mental Health Unit, Centre for Mental Health, Melbourne School of Population and Global Health, The University of Melbourne, Melbourne, Australia

**Keywords:** Suicide prevention, Mental health first aid, Helping behaviour, Assistance, Gatekeeper training, Sri Lanka

## Abstract

**Background:**

Sri Lanka has one of the highest suicide rates in the world. Gatekeeper programs aimed at specific target groups could be a promising suicide prevention strategy in the country. The aim of this study was to develop guidelines that help members of the public to provide first aid to persons in Sri Lanka who are at risk of suicide.

**Methods:**

The Delphi method was used to elicit consensus on potential helping statements to include in the guidelines. These statements describe information members of the public should have and actions they can take to help a person who is experiencing suicidal thoughts. An expert panel, comprised of mental health and suicide experts in Sri Lanka, rated each statement. The panellists were encouraged to suggest any additional action that was not included in the original questionnaire and, in particular, to include items that were culturally appropriate or gender specific. Responses to open-ended questions were used to generate new items. These items were included in the subsequent Delphi rounds. Three Delphi rounds were carried out. Statements were accepted for inclusion in the guidelines if they were endorsed (rated as essential or important) by at least 80 % of the panel. Statements endorsed by 70–79 % of the panel were re-rated in the following round. Statements with less than 70 % endorsement, or re-rated items that did not receive 80 % or higher endorsement were rejected.

**Results:**

The output from the Delphi process was a set of endorsed statements. In the first round questionnaire 473 statements were presented to the panel and 58 new items were generated from responses to the open-ended questions. Of the total 531 statements presented, 304 were endorsed. These statements were used to develop the suicide first aid guidelines for Sri Lanka.

**Conclusion:**

By engaging Sri Lankans who are experts in the field of mental health or suicide this research developed culturally appropriate guidelines for providing mental health first aid to a person at risk of suicide in Sri Lanka. The guidelines may serve as a basis for developing training for members of the public to provide mental health first aid to persons at risk of suicide as part of Sri Lanka’s suicide prevention strategy.

**Electronic supplementary material:**

The online version of this article (doi:10.1186/s13033-016-0085-3) contains supplementary material, which is available to authorized users.

## Background

Suicide is a significant social and public health problem, yet it is largely preventable [1]. Every year there are more than 800,000 suicides around the world [1, 2]. By the year 2020, suicide is estimated to contribute more than 2 % to the global burden of disease (1). South East Asia and the Western Pacific regions account for more than half of the suicides that occur globally every year [3]. The world’s most vulnerable populations, including the young, the elderly and the socially isolated, are at particular risk of suicide. A high burden of disease is attributable to suicide in low- and middle-income countries (LMICs), which are often ill-equipped to meet the mental health needs of their populations, with services that are inadequately developed, often difficult to access and of poor quality [1].

Among the many suicide prevention strategies that have been developed some have shown excellent results. However, prevention efforts are more limited in LMICs than in high-income countries despite the fact that most suicides (75 %) occur in LMICs [4, 5]. Studies evaluating prevention efforts in LMICs are few. Most studies of prevention programs have been conducted in high-income countries and their relevance to LMICs is unknown (5). Although evidence on which suicide prevention strategies are most effective is limited, a few promising approaches have been identified. In a 2005 study, a panel of 15 suicide experts identified five major areas of prevention: education and awareness programs for the general public and professionals, screening methods for persons at high risk, treatment of psychiatric disorders, restricting access to lethal means, and changing media reporting of suicide [6]. Available evidence suggests that physician education, restriction of means and gatekeeper education are promising interventions [6].

Sri Lanka has one of the highest suicide rates in the world, 25 per 100,000 in 2005 [7]. These rates, although still high, are a substantial improvement from 1995 where the suicide rate peaked at 47 per 100,000 [7]. The reduction that has been achieved is reflective of the fact that Sri Lanka is one of the few LMICs that have formulated a specific national suicide prevention plan and taken action on suicide prevention [4]. Among the actions implemented was the decriminalisation of suicide in 1998 [7]. The decline in rates closely followed the banning of highly toxic pesticides in the country [7]. Most of Sri Lanka’s suicide prevention strategies have been focussed on restricting access to lethal means, such as pesticides, as acute poisoning is the most common method of suicide in the country [7, 8]. While withdrawing or restricting a number of pesticides, and initiating plans for safe use of pesticides through programs such as integrated pest management [9], have been effective [7, 10, 11], further areas of suicide prevention/intervention must be developed, as the current suicide rate in the country is still high. Suicide prevention interventions such as education and awareness programs for the general public and professionals could be beneficial in Sri Lanka, especially programs referred to as gatekeeper training [12].

Gatekeeper training involves the training of people in the community to recognise and identify those who are at risk of suicide and assist them in receiving appropriate care [13, 14]. In essence, gatekeepers ‘open the gate to help’ for those at risk of suicide [13]. The best suited to act as gatekeepers are family members and friends due to their close relationship with the person at risk [13]. However, gatekeepers may also include professionals who are in frequent contact with potentially vulnerable populations, such as public health officers (public health inspectors and midwives in Sri Lanka), religious leaders, first responders, persons employed in schools, prisons and military, and caregivers [6].

Several gatekeeper training methods are available as train-the-trainer programs [13]. Examples of such programs include the applied suicide intervention skills training (ASIST) by Living Works; Question, Persuade and Respond (QPR) and Yellow Ribbon International (YRI) for suicide prevention [13, 15]. Although these programs are being implemented in many countries, there is a lack of conclusive evidence of effectiveness [16]. Most of the studies and evaluations that have been conducted have been with school staff, in workplaces and the military [6, 13, 15]. Studies of the effectiveness of gatekeeper training have also been conducted with indigenous and Aboriginal communities [13, 14]. Uncontrolled evaluations of ASIST and QPR have found a positive impact on self-reported preparedness and observer-rated skills in suicide intervention [13]. A randomised controlled trial among school staff in the United States showed an increased self-reported knowledge of suicide risk but had little impact on gatekeeper behaviour during follow up [13]. A similar outcome was seen in a study done in a remote First Nations community [13]. However, studies done on gatekeeper training in the Norwegian Army and the US Air force have reported success in lowering suicide rates [6]. Further research is necessary to evaluate effectiveness of gatekeeper training in preventing suicide. Nonetheless, there is enough evidence to support a conclusion that gatekeeper training is effective in changing attitudes and behaviours, with increased knowledge about suicide and reduction in negative attitudes towards people who are at risk of suicide [13, 15]. Mental health first aid (MHFA) training courses, which have been delivered in all states in Australia and 23 other countries, have shown similar results [17, 18]. Evaluations of these programs have consistently shown that MHFA training is associated with improved knowledge of mental illnesses and their treatment, knowledge of appropriate first aid strategies, and confidence in providing first aid to individuals with mental illness [19], that these benefits are sustained over time [20] and that it is possible to reach very large numbers of the general population [21]. Some studies have also shown improved mental health in those who attended the training, decrease in stigmatising attitudes and increase in the amount and types of support provided to others [20, 22]. Such change in knowledge, attitudes, confidence and behaviour could prove beneficial in communities where mental illness and suicide are stigmatised, for example among communities in Sri Lanka [23].

The availability of guidelines that would assist members of the public in how they should provide first aid to a person at risk of suicide could form the basis for the development and evaluation of gatekeeper training programs or other mental health first aid training courses. An obstacle to the development of such guidelines is the limited systematic evidence to guide the content of suicide gatekeeper training. In such circumstances consensus methods such as the Delphi process can be useful to develop appropriate guidelines [22]. This method embodies the principle of practice-based evidence [22]. Results relevant to the local population and culture can be attained by harnessing the expertise of professionals or people working in the area [22]. This method is relatively inexpensive and, as Delphi group members do not need to meet, the study can be done using the internet [24–26]. The Delphi method has been widely used in health research, [27, 28] and has been used to develop suicide first aid guidelines for English-speaking countries [29, 30]. This method has also been used to develop suicide first aid guidelines for Asian countries such as India, Japan and the Philippines and for people from immigrant and refugee background [31–34]. There were similarities and differences among the guidelines produced for each of the Asian countries, therefore this project was undertaken to develop country-specific suicide first aid guidelines for Sri Lanka.

The aim of the project was to produce guidelines for members of the public providing first aid for a person in Sri Lanka who is having suicidal thoughts or displaying suicidal behaviours. No study of this kind has been conducted previously in Sri Lanka. In this study, the role of a mental health first aider was defined as “someone who helps a person who is developing a mental health problem or is in a mental health crisis”.

## Methods

The study had three phases: a literature search, questionnaire development and the Delphi process.

### Systematic search for possible suicide first aid actions in the literature

As part of a project to revise previous work [29] done to develop suicide first aid guidelines for developed English-speaking countries a systematic search of relevant literature was carried out by the MHFA team [29, 30]. Briefly, this search identified information about how to determine whether someone is having thoughts of suicide and what possible first aid actions can be undertaken. The items developed by Ross et al. [29, 30] were used as the basis for development of the first round survey used in this project.

### Questionnaire development

The questionnaire for English-speaking countries [30] was developed from analysing the literature mentioned above and creating statements that suggest a potential first aid action (e.g. what the first aider should do or should not do) or statements that suggest what a first aider should know. These statements were grouped into common categories: identification of suicide risk, assessing seriousness of risk, initial assistance, talking with the suicidal person, no-suicide contracts, ensuring safety, passing time during the crisis, what the first aider should know, confidentiality and adolescent-specific statements [30]. A working group was convened to ensure that the questionnaire did not include items that might be difficult to understand, repetitive or ambiguous.

The questionnaire developed for English-speaking countries had 436 items, each describing a potential action by a first aider that could be presented to the panel for rating [30]. This initial questionnaire was modified for the current study with items generated in recent studies on suicide prevention for people from immigrant and refugee background and in Asian countries, in order to improve its cultural relevance [31, 33, 34]. The initial questionnaire for Sri Lanka thus contained 473 first aid action items, plus six questions on participants’ socio-demographics and experience/training. Open-ended questions were included after each category, to give participants the opportunity to suggest culturally specific actions that could be used to generate further items for subsequent rounds of the Delphi process. An open-ended question was also included to gather gender-specific items regarding suicide in Sri Lanka. The questionnaire was translated to both Sinhala and Tamil, the two most common languages spoken in Sri Lanka.

### Forming the expert panel

To qualify as an ‘expert’, participants were required to have knowledge about suicide through their experience as a mental health professional or as a suicide prevention lived experience advocate. Participants were recruited in several ways. Clinical experts in medicine, psychiatry, public health, social work and psychology working in Sri Lanka were identified by the authors and invited to participate. Professionals who had published on suicide in Sri Lanka were also invited to be part of the panel. Attempts were made to recruit people with lived experience (i.e. people who have experienced suicidal ideation or made a suicide attempt in the past) through advocacy organisations in the country, but this proved to be unsuccessful. The main difficulty conveyed by the advocacy organisations were concerns about confidentiality and the stigma attached to suicide in Sri Lanka.

Invitation letters were sent using the web-based SurveyMonkey [35], and further information about the study was provided through a web page link, which contained the plain language statement (PLS) in English, Sinhala and Tamil. The invitation letter also asked the participants to recommend or nominate colleagues who could be eligible as panel members. The email also contained the link for the first round of the English version of the questionnaire and participants were advised to contact the researchers if they preferred to receive the Sinhala or Tamil versions of the questionnaire, or printed copies of the survey. No participants opted for these alternatives.

In Round 1, the questionnaire derived from the process described above was sent to 77 potential panel members. It provided instructions and definitions to guide the panel members. Panel members were asked to rate each statement by indicating their level of agreement that the statements should be included in guidelines for a first aider who is helping a suicidal person. The 5-point Likert scale was: essential, important, do not know/depends, unimportant and should not be included.

Participants were asked to indicate whether they considered any item in the first round questionnaire to be culturally irrelevant or unacceptable, or not feasible because of limitations in the health system and resources in the country. Furthermore, after each category participants were asked to suggest any additional item that was not included in the initial questionnaire. This encouraged culturally and gender specific material to be introduced. Suggestions made by the panel members in response to the open-ended questions were analysed and used to construct new items for the Round 2 questionnaire.

Round 1 responses were analysed to calculate the percentage of the panel who rated an item as either “essential” or “important”. Items that were endorsed by 80 % or more of the panel were accepted for inclusion in the guidelines. Statements rated by 70–79 % of the members of the panel as essential or important were re-rated in the subsequent round of the Delphi process. Statements that were rated by less than 70 % of the panel members as essential or important were rejected. These are the same consensus thresholds for inclusion and re-rating used in previous Delphi studies [32–34].

The Round 2 questionnaire consisted of new items that were generated from the open-ended questions in Round 1 and items from Round 1 to be re-rated. Participants were asked to rate the items in the Round 2 questionnaire. At the end of the round, percentages were analysed and items that reached the 80 % consensus criterion were accepted for the guidelines. Items that were re-rated in Round 2 and were below 80 % consensus were rejected. Items that were generated from open-ended questions in Round 1 and achieved only 70–79 % consensus in Round 2, were included to be re-rated in Round 3. As in Round 2, items that reached consensus were included in the guidelines and the rest were rejected.

## Results

### The expert panel

In Round 1, of the 77 potential expert panel members invited, 31 endorsed to participate in the study and returned the questionnaire. Of these, 17 participants’ questionnaires were only partially completed and were not included in the quantitative analysis. The 14 respondents who submitted fully completed questionnaires constituted the expert panel. From the 14 members in Round 1, 12 panel members completed the Round 2 and 3 questionnaires. All panel members were currently working or had recently worked in Sri Lanka. There were eight psychiatrists, two psychologists/counsellors, a social worker, a registrar in psychiatry, a medical officer of mental health and a community physician. The panel consisted of six males and eight females. Eight of the participants were in the age range of 40–49 years, four participants were aged 30–39 years and two were aged 51–56 years. Panel members worked in Colombo (Western Province), Kilinochchi, Mullaitivu, and Mannar (Northern Province), Kalmunai and Valaichenai (Eastern Province), Galle (Southern Province) and Matale (Central Province).

### Items endorsed

**Fig. 1:**
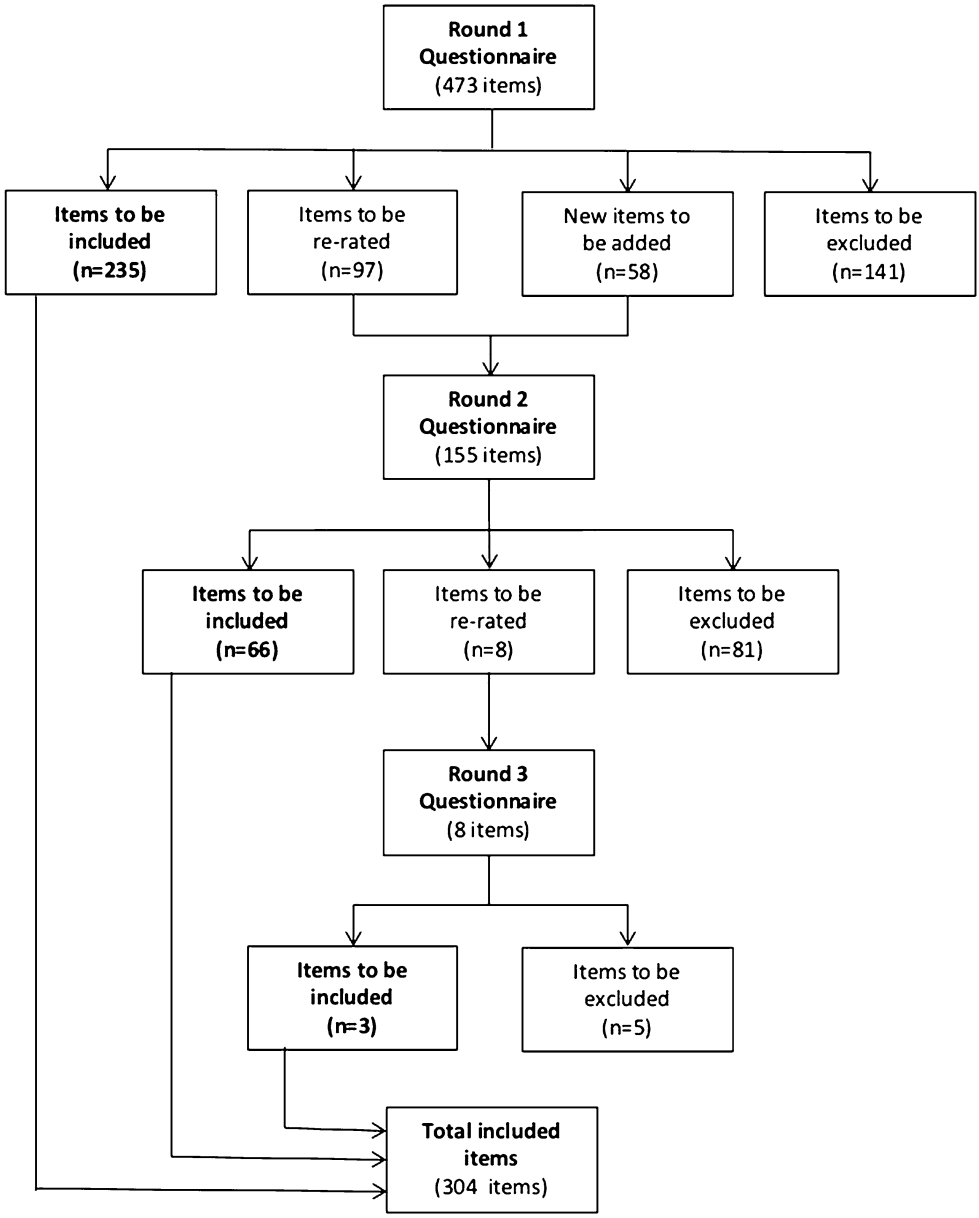
Items endorsed, rejected and re-rated at each round

Figure 1 shows the rates of endorsement, rejection and re-rating of items in each Delphi round. In the first round, of the 473 items included, 235 were endorsed, 141 were rejected and 97 met the criteria for re-rating. An additional 58 new items were created from the panel members’ responses to the Round 1 open-ended questions. Of the 155 items included in Round 2 66 were endorsed, 81 were rejected, and eight met criteria for re-rating. Of the eight items included in the questionnaire in Round 3, three were endorsed and five were rejected. At the end of Round 3, of the total 531 statements rated by the panel, 304 were endorsed.

The following are some examples of the new items that were generated from suggestions by the panel and that were included in the Round 2 questionnaire:

- An important warning sign for suicide is if a person is expressing interest in renouncing lay life and joining a religious institution (e.g. going into robes, entering priesthood or becoming a nun).
- The first aider should be aware of the stigma associated with suicide and that this might discourage the suicidal person from disclosing suicidal thoughts.
- The first aider should be aware that the safety plan should be developed taking into account the suicidal persons sociocultural background.
- The first aider should be aware of different risk factors for suicidal women such as domestic violence.
- The first aider should be aware that with females, it is important to discuss relationships issues, sexual interactions and related concerns.

A number of responses to the Round 1 open-ended questions did not meet criteria for creation of a new item (i.e. they did not fit the definition of first aid or did not suggest a clear action) or were simply comments or suggestions. The following are examples of the comments and suggestions that did not generate new items:

- In Sri Lanka the group of people who attempt suicide with brief planning while not having a mental illness is quite sizable (…).
- Emergency hot lines are not available in most of the areas. So we need to utilize lower resources in our area.
- Sri Lankan people are not yet familiar with suicide hotlines and 24 h phone help when in distress. It will take a long time for this facility to develop in rural areas in Sri Lanka (…).
- Most of the adolescents who inflict self-harm are not suicidal and also don’t have any mental illness.

Table 1 shows examples of statements endorsed for inclusion in the writing of the guidelines and the Delphi round in which the item was endorsed.

**Table 1:** Examples of statements endorsed for inclusion in the guidelines

Items	Round
Section 1: Identification of suicide risk	
The first aider should not assume that the person will get better without help	1
The first aider should be aware that if a person is suicidal, asking them about suicidal thoughts will not increase the risk that they will act on these	2
The first aider should be aware that a person who self-harms may not be suicidal. If the first aider is unsure whether noticed injuries are from self-harm or from a suicidal attempt, the first aider should ask the person directly	2
Section 2: Assessing seriousness of the suicide risk	
The first aider should find out if the suicidal person has already taken steps to secure the means to end their life	1
The first aider should ask the suicidal person if they have ever made a suicide attempt in the past	1
The first aider should be aware that the method and specific modality (e.g. quantity of poison) the suicidal person plans to use indicate the seriousness of the suicidal intention	2
Section 3: Initial Assistance to suicidal people	
The first aider should remain calm and in control when communicating with a suicidal person	1
The first aider should work collaboratively with the suicidal person to ensure their safety, rather than acting alone to prevent suicide	1
The first aider should provide the suicidal person with information and resources about where they can seek help	1
Section 4: Talking to a suicidal person	
The first aider should ask open questions to find out more about the suicidal thoughts and feelings and the problems behind these	1
The first aider needs to allow the suicidal person to talk about their reasons for wanting to die	1
The first aider should reassure the suicidal person that it is not a crime, a sin or shame to feel suicidal	2
Section 5: Safety planning with suicidal people	
The first aider should engage the suicidal person to the fullest possible extent in decisions about a safety plan	1
If the suicidal person agrees, the first aider should involve someone the person trusts in developing the safety plan such as a friend, family member, mental health professional or a religious or spiritual leader	2
Section 6: Ensuring safety for suicidal people	
The first aider should help the suicidal person decide who they can contact if they become suicidal again	1
If the suicidal person agrees, the first aider should inform community mental health workers or psychosocial workers in the area about the situation	2
Section 7: Passing time during a crisis	
The first aider should develop a list with the suicidal person of other things they can do distract themselves	1
Section 8: What the first aider should know in providing suicide first aid	
The first aider should be aware that openly talking about suicidal thoughts and feelings can save a life	1
Section 9: Confidentiality	
The first aider should treat the suicidal person with respect and involve them in decisions about who else knows about the suicidal crisis	1
Section 10: Specific to adolescents	
If the suicidal person is a minor, the first aider must make their significant elders aware of the person’s intention to kill themselves	2
Section 11: Gender specific	
The first aider should be aware of different risk factors for a suicidal women such as domestic violence	2
The first aider should be aware that some males may be less likely to express their emotions and open up about suicidal intentions	2
The first aider must be aware that Sri Lankan men may not openly disclose previous suicide attempts and may instead state, for example, that they had an “accidental overdose of medication or poison”	3

To be usefully communicated, the list of action statements on which there was consensus were used to write, in narrative text form, the Suicide First Aid Guidelines for Sri Lanka. The draft guidelines were sent to the expert panel for final comments before preparing the final guidelines. The guidelines (see Additional file 1) provide knowledge about major warning signs for suicide in Sri Lanka, how to get ready to approach the suicidal person, how to talk to them, how to recognize the urgency of the situation, how to keep the person safe, who and how to approach to receive help, how to manage confidentiality and threatening situations, and how to take care of oneself as first aider. In addition to these sections, they also contain a box with key information and data about suicide in Sri Lanka, a box about how to deal with communication difficulties (which contains also gender-specific actions), listening tips, ‘not to do’ actions, and safety plan.

## Discussion

The aim of this project was to identify consensus among professionals on first aid actions that members of the public can take to help a suicidal person in Sri Lanka. A total of 304 first aid statements achieved consensus among the panel of experts.

In response to the open-ended questions comments were made by panel members regarding the lack of mental health resources in some rural areas in Sri Lanka, especially suicide hotlines. There is also a lack of awareness in the community of the existence of such hotlines where they do exist [12]. Suicide hotlines are mostly run by non-government organisations such as Sumithrayo [36], and CCCline, founded in 2009 by the CCC Foundation. A great deal of work has been undertaken to expand such services to rural areas [4, 8, 12]. Compared to other countries where similar studies have been undertaken [32–34], advocacy and support organizations for suicide in Sri Lanka are limited. These guidelines could be used as an additional training tool by mental health and suicide prevention organisations for training of non-mental health professional staff and volunteers.

As there is evidence in the literature that suicide in Sri Lanka is more common among adolescents, educating potential gatekeepers in schools and universities on how to help a young person at risk could be most useful. A focus group conducted with students about their perspectives on suicidal behaviours identified that teachers could be potential gatekeepers as they have significant and positive contact with young people and most students viewed them as accessible [12]. Religious leaders could also have an important role in suicide prevention in the country, as religion plays an important part in Sri Lanka society [23]. Some panel members commented that changes in a person’s religious beliefs, with either an increased or decreased interest in their faith, may indicate increased risk of suicide. Educating religious leaders about warning signs of suicide and how to identify and help a person at risk of suicide could prove helpful. Gatekeeper training programs for teachers [12] and religious leaders based on these guidelines may prove to be an effective component of a broader suicide prevention strategy in Sri Lanka. While the guidelines constitute a basis for the development of training they may need to be further tailored to the specific religious and cultural factors in different regions in Sri Lanka, as studies have shown a difference in attitudes to and beliefs about suicide, and suicide rates, among different religious and cultural groups [37, 38].

Domestic violence, alcohol abuse by a partner and relationship issues are important risk factors for suicide among females in Sri Lanka [39]. Therefore, when developing suicide prevention training programs, these specific issues must also be addressed, within the specific socio- cultural context. For example, gatekeepers must know of services available to assist a person experiencing domestic violence and refer the person to such services. They could also be trained to help women develop safety plans to keep themselves and their children safe during violent episodes [39] which may provide women with viable alternatives to suicidal behaviour and also support them to take effective action in the context of domestic violence.

### Future directions

The guidelines should be translated to Sinhala and Tamil and a visually simplified version for low-literacy population (e.g. an infographic as developed for the guidelines for people from immigrant and refugee background see [31]) should be developed. The various versions of the guidelines should then be distributed freely among the community and institutional settings to increase awareness about suicide and possible prevention strategies. The guidelines can be used as a basis for the development of training programs and can be implemented for specifically vulnerable groups such as adolescents and women experiencing domestic violence. To ensure that suicide prevention training is relevant to specific communities training programs should be developed in consultation with community members within the area so that ethnic, cultural and religious differences regarding suicide, for example within Sinhala and Tamil communities, are taken into consideration. In areas that are less developed, and that have limited resources, these guidelines could be used also by primary health workers, midwives and nurses. Any training programs developed with the use of these guidelines should be rigorously evaluated to determine impact on suicide prevention.

## Limitations

A limitation of this study is the small number of panel members, which reduces reliability in estimating consensus, although some authors recommend 10–18 experts on a Delphi panel [25]. The presence of heterogeneous or homogeneous samples also influences the decision concerning the optimum number of panel members. For a homogeneous group, 10–15 people can be sufficient [40]. However, further studies should aim to recruit panel members with lived experience. Developing a methodology in which the concerns of the people with lived experience regarding confidentiality and stigma attached to suicide are addressed could result in a wider representation of those with such experience.

Although the questionnaire was made available in Sinhala and Tamil as well as English, only the English version of the questionnaire was used, since none of the respondents asked for a Sinhala or Tamil version of the questionnaire. It is possible that there would have been more culturally specific responses if members of the panel had requested to use the questionnaire in their native language.

## Conclusion

Members of the public and the large number of minimally trained health workers in Sri Lanka, such as public health inspectors, [41, 42] can play an important role in preventing suicide. Engaging Sri Lankans who are experts in the field of mental health and suicide, developing suicide first aid guidelines for members of the public, and training programs based on these, might contribute to community capacity to prevent suicide. It is also necessary to increase awareness regarding suicide and to decrease the stigma associated with suicide and suicidal behaviours.

The free availability of these guidelines may expand opportunities for the public to engage in better informed discussion about suicide as a major public health and social issue, learn basic mental health first aid actions to assist the suicidal person, and learn how to take such actions when needed. Such outcomes may contribute to more effective suicide prevention in Sri Lanka.

## Additional file

10.1186/s13033-016-0085-3 Suicide first aid guidelines for Sri Lanka.

## Abbreviations

ASIST: applied suicide intervention skills training
LMICs: low- and middle-income countries
MHFA: Mental Health First Aid
PLS: plain language statement
QPR: question, persuade and respond
YRI: yellow ribbon international

## References

[1] World Health Organization. Public health action for the prevention of suicide: a framework. World Health Organization; 2012.

[2] Suicide prevention (SUPRE). http://www.who.int/mental_health/prevention/suicide/suicideprevent/en/. Accessed 7 Mar 2016.

[3] WHO: Suicide and suicide prevention in Asia. In: World Health Organization; 2008: 118.

[4] Vijayakumar L, Pirkis J, Whiteford H (2005). Suicide in developing countries (3): prevention efforts. Crisis.

[5] World Health Organization. Preventing suicide: a global imperative. Geneva: World Health Organization; 2014.

[6] Mann J, Apter A, Bertolote J (2005). Suicide prevention strategies: a systematic review. JAMA.

[7] Gunnell D, Fernando R, Hewagama M, Priyangika WD, Konradsen F, Eddleston M (2007). The impact of pesticide regulations on suicide in Sri Lanka. Int J Epidemiol.

[8] Konradsen F, Hoek W, Peiris P (2006). Reaching for the bottle of pesticide–a cry for help. Self-inflicted poisonings in Sri Lanka. Soc Sci Med.

[9] SP-IPN Secretariat. Incorporating integrated pest management into national policies. In: Croydon, editor. Ibadan: International Institute of Tropical Agriculture; 2008.

[10] Mohamed F, Manuweera G, Gunnell D, Azher S, Eddleston M, Dawson A, Konradsen F (2009). Pattern of pesticide storage before pesticide self-poisoning in rural Sri Lanka. BMC public health.

[11] Weerasinghe M, Pieris R, Eddleston M, Hoek W, Dawson A, Konradsen F (2008). Safe storage of pesticides in Sri Lanka—identifying important design features influencing community acceptance and use of safe storage devices. BMC public health.

[12] Ratnayake R, Links P (2009). Examining student perspectives on suicidal behaviour and its prevention in Sri Lanka. Int J Soc Psychiatry.

[13] Sareen J, Isaak C, Bolton SL, Enns MW, Elias B, Deane F, Munro G, Stein MB, Chateau D, Gould M (2013). Gatekeeper training for suicide prevention in First Nations community members: a randomized controlled trial. Depress Anxiety.

[14] Clifford A, Doran C, Tsey K (2013). A systematic review of suicide prevention interventions targeting indigenous peoples in Australia, United States, Canada and New Zealand. BMC public health.

[15] Tompkins TL, Witt J, Abraibesh N (2010). Does a gatekeeper suicide prevention program work in a school setting? evaluating training outcome and moderators of effectiveness. Suicide Life Threat Behav.

[16] Isaac M, Elias B, Katz LY, Belik S-L, Deane FP, Enns MW, Sareen J (2009). Gatekeeper training as a preventative intervention for suicide: a systematic review. Can J Psychiatry.

[17] Mental Health First Aid: Our Global Impact. https://mhfa.com.au/our-impact/our-global-impact. Accessed 7 Mar 2016.

[18] Mental Health First Aid: Course Evaluations. https://mhfa.com.au/research/mhfa-course-evaluations-swedishev. Accessed 7 Mar 2016.

[19] Mendenhall AN, Jackson SC, Hase S (2013). Mental Health First Aid USA in a Rural Community: perceived Impact on Knowledge, Attitudes, and Behavior. Soc Work Mental Health.

[20] Svensson B, Hansson L (2014). Effectiveness of mental health first aid training in sweden. a randomized controlled trial with a six-month and two-year follow-up. PLoS One.

[21] Jorm AF, Kitchener BA (2011). Noting a landmark achievement: mental health first aid training reaches 1% of Australian adults. Aust N Z J Psychiatry.

[22] Minas H, Jorm AF (2010). Where there is no evidence: use of expert consensus methods to fill the evidence gap in low-income countries and cultural minorities. Int J Ment Health Syst.

[23] Marecek J (1998). Culture, gender, and suicidal behavior in Sri Lanka. Suicide Life Threat Behav.

[24] Linstone HA, Turoff M. The Delphi method: techniques and applications. Boston: Addison-Wesley; 2002.

[25] Okoli C, Pawlowski SD (2004). The Delphi method as a research tool: an example, design considerations and applications. Inf Manag.

[26] Powell C (2003). The Delphi technique: myths and realities. J Adv Nurs.

[27] Hasson F, Keeney S, McKenna H (2000). Research guidelines for the Delphi survey technique. J Adv Nurs.

[28] Jorm A (2015). Using the Delphi expert consensus method in mental health research. Aust N Z J Psychiatry.

[29] Kelly CM, Jorm AF, Kitchener BA, Langlands RL (2008). Development of mental health first aid guidelines for deliberate non-suicidal self-injury: a Delphi study. BMC psychiatry.

[30] Ross AM, Kelly CM, Jorm AF (2014). Re-development of mental health first aid guidelines for suicidal ideation and behaviour: a Delphi study. BMC psychiatry.

[31] Colucci E, Jorm AF, Kelly CM, Too LS, Minas H: Mental health first aid guidelines for helping a suicidal person from an immigrant and refugee background: A Delphi consensus study. 2014 **(Under Submission)**.

[32] Colucci E, Kelly CM, Minas H, Jorm AF, Chatterjee S (2010). Mental Health First Aid guidelines for helping a suicidal person: a Delphi consensus study in India. Int J Ment Health Syst.

[33] Colucci E, Kelly CM, Minas H, Jorm AF, Nadera D (2010). Mental health first aid guidelines for helping a suicidal person: a Delphi consensus study in the Philippines. Int J Ment Health Syst.

[34] Colucci E, Kelly CM, Minas H, Jorm AF, Suzuki Y (2011). Mental health first aid guidelines for helping a suicidal person: a Delphi consensus study in Japan. Int J Ment Health Syst.

[35] Survey Monkey. https://www.surveymonkey.com/.

[36] Sri Lanka Sumithrayo. http://www.srilankasumithrayo.org/. Accessed 7 Mar 2016.

[37] Simpson ME, Conklin GH (1989). Socioeconomic development, suicide and religion: a test of Durkheim’s theory of religion and suicide. Soc Forces.

[38] Bertolote JM, Fleischmann A (2002). A global perspective in the epidemiology of suicide. Suicidology.

[39] Marecek J (2006). Young women’s suicide in Sri Lanka: cultural, ecological, and psychological factors. Asian J Couns.

[40] Skulmoski G, Hartman F, Krahn J (2007). The Delphi method for graduate research. J Inf Technol Educ.

[41] Kakuma R, Minas H, van Ginneken N, Dal Poz MR, Desiraju K, Morris JE, Saxena S, Scheffler RM (2011). Human resources for mental health care: current situation and strategies for action. Lancet.

[42] Minas H (2015). A mental health human resources strategy for Sri Lanka. Sri Lanka J Psychiatry.

