# Supplementary material for: Suicide first aid guidelines for Sri Lanka: a Delphi consensus study
Source: Int J Ment Health Syst. 2016 Aug 30;10(1):53. doi: 10.1186/s13033-016-0085-3 (PMC5004258; doi:10.1186/s13033-016-0085-3)
Supplement: Supplementary file 1 — 10.1186/s13033-016-0085-3 Suicide first aid guidelines for Sri Lanka. [file 13033_2016_85_MOESM1_ESM.pdf]

Suicide First Aid Guidelines for Sri Lanka

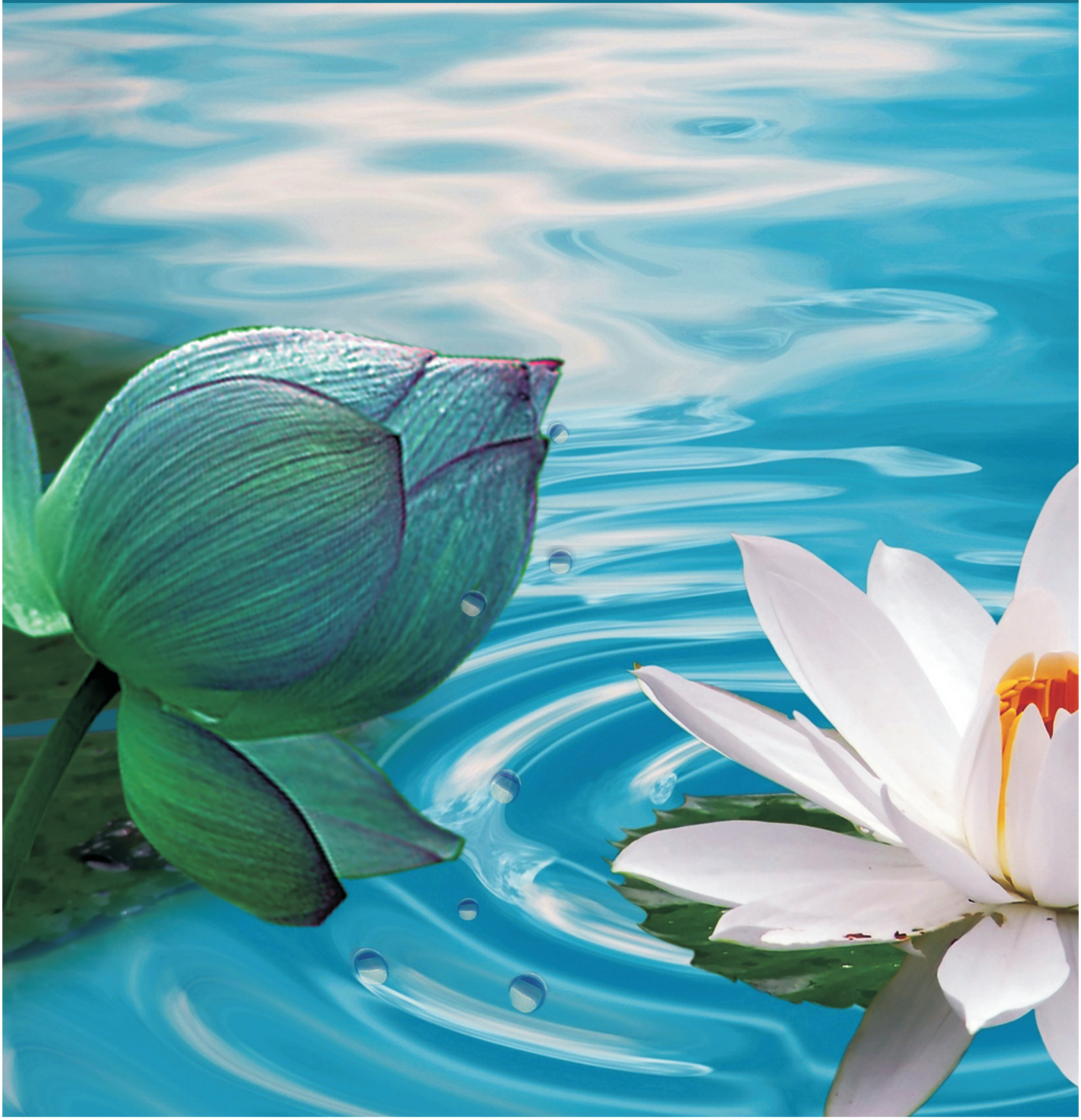

Suicide can be prevented. Most suicidal people do not want to die, they just do not want to live with the pain they are feeling. Helping a suicidal person talk about their thoughts and feelings can help save a life. Do not underestimate your abilities to help a suicidal person, even to save a life.

FACTS ABOUT SUICIDE IN SRI LANKA

- Sri Lanka has one of the highest suicide rates in the world with an incidence of 25 per 100,000 in 2005.
- Males have a higher rate of suicide than females: in 2005 the suicide rates were 37.3 per 100,000 in males and 9.7 per 100,000 in females. However, Sri Lanka's rate of female suicide is second only to China.
- A study in 2006 of all deaths given a verdict of 'suicide' by the coroner during a one year period showed that during that time the nearly two-thirds of the victims were below the age of 40 years.
- Pesticide poisoning is the most common method used for suicide in Sri Lanka, followed by self-burning, hanging, drowning or jumping in front of a train.

MAIN RISK FACTORS :

- Socio-demographic characteristics:
  - ✓ Young age
  - ✓ Low educational level
- Negative life events:
  - ✓ Having ended an emotional relationship
  - ✓ Conflict with family members
  - ✓ Domestic violence
  - ✓ Failing an examination
  - ✓ Unemployment
  - ✓ Debt
  - ✓ Being exposed to suicide by a close relative
- Psychological variables:
  - ✓ Alcohol dependence
  - ✓ Mental illness.

1. Gunnell D, Fernando R, Hewagama M, Priyangika WD, Konradsen F, Eddleston M. The impact of pesticide regulations on suicide in Sri Lanka. International Journal of Epidemiology. 2007;36(6):1235-42.
2. Fernando R, Hewagama M, Priyangika WDD, Range S, Karunaratne S. Study of suicides reported to the Coroner in Colombo, Sri Lanka. Medicine, Science and the Law. 2010;50(1):25-8.
3. Maracek J. Young women's suicides In Sri Lanka: Cultural, ecological and psychological factors. Asian Journal of Counselling. 2006;13(1):63-92.
4. van der Hoek W, Konradsen F. Risk factors of acute pesticide poisoning in Sri Lanka. Tropical Medicine and International Health. 2005;10(6).

How can I tell if someone is feeling suicidal?

A suicidal person may not ask for help directly, but they are likely to show certain warning signs. It is really important that you are able to recognise some of the warning signs for suicide.

Signs a person might be suicidal

A person may show a big change in mood, behaviour or appearance, for example:

- Expressing, in words or actions:
  - hopelessness or feeling that their life is worthless
  - having no reason to live or no purpose in life
  - no interest in or plans for the future
  - strong sense of feeling alone and cut off
  - feelings of guilt or shame, or belief of being a burden to others (e.g. saying “others will be better off without me” or “I have brought shame on my family”)
  - Feelings of extreme dislike or hatred of oneself
- Withdrawing from friends, family or the community
- Suddenly becoming very sad or a sad person becomes much more depressed.

A person may threaten to hurt or kill themselves, or say that they wish to die, verbally (speaking) or in writing. This may be direct but sometimes is subtle and not obvious. Watch for:

- Talking or writing about death, dying or suicide (including making unexpected jokes about these topics or leaving a suicidal note, poem or letter).
- Looking for a way to kill themselves (e.g. trying to get pills or poisons, weapons or othermeans), including asking for information about possible suicide methods (e.g. “will a bottle of this medicine kill me?”). Be aware that people may use different methods to carry out suicide, so pay attention to the presence of any sort of things that could be used for suicide (e.g. sharp objects, poisons - such as pesticides and seeds, and kerosene).
- Expressing in words or actions that they feel trapped, there is no way out, or that they are unable to find an alternative solution to their problem and that suicide is the only solution to their problems.

A person may behave in ways that are life-threatening or dangerous, for example:

Stopping life-saving medical treatments or medications.

A person may try to set their affairs and relationships in order, for example:

- Giving away valued possessions.
- Asking others to take on responsibility for the care of people or pets.
- Contacting people (e.g. family members or other people they have not spoken to in a long time) to say goodbye, make peace or ask for forgiveness.

People may show one or many of these signs, and some may not show any signs on this list.

If you have noticed some of these warning signs and you are concerned a person may be at risk of suicide, you need to talk to them about your concerns. If you are not sure whether what you have noticed is a reason to be alarmed, you could ask someone who knows the person better than you, if they are worried too.

Getting ready to approach the person

Be aware of your own attitudes, think about how you feel about suicide and how this will impact on your ability to help (e.g. belief that suicide is wrong or that it is an acceptable option). If the person is from a different cultural or religious background to you, remember that they might have beliefs and attitudes about suicide that are different from your own. So it may help to learn more about the common cultural (including spiritual and religious) beliefs about suicide amongst the people who you have frequent interactions with.

Choose a private place to talk to the person. If you feel you are unable to ask the person about suicidal thoughts, find someone else who can.

Making the approach

Act quickly if you think someone is considering suicide. Even if you only have a mild suspicion that the person is having suicidal thoughts, you should still approach them.

Tell the person you are concerned about them and describe the behaviours that are worrying you. Be patient and give them time to talk about their negative feelings before asking about suicidal thoughts.

Be aware that the person may not want to talk with you, or you might have difficulty connecting with them. If this happens, you should offer to help them find someone else to talk to.

Asking about thoughts of suicide

Anyone could have thoughts of suicide. If you think someone might be having suicidal thoughts, you should ask that person directly. Unless someone tells you, the only way to know if they are thinking about suicide is to ask. For example, you could ask:

"Are you having thoughts of suicide?" or

"Are you thinking about killing yourself?"

Be mindful of how you ask someone - the words you use are very important. You should not ask about suicide in a judgmental way for example don't say "You're not thinking of doing anything stupid, are you?".

See Box 1 Dealing with communication difficulties

Sometimes people don't want to ask directly about suicide because they think they will put the idea into the person's head. This is not true. If a person is suicidal, asking them about suicidal thoughts will not increase the risk that they will do it. Instead, asking the person about suicidal thoughts will give them the chance to talk about their problems and show them that somebody cares.

Even though it is common to feel panic or shock when someone says they are thinking about suicide, it is important to not react negatively, e.g. show judgment, shock, panic or anger. Do your best to appear calm, confident and empathic, as this may have a reassuring effect on the suicidal person.

Box 1

Dealing with communication difficulties

It is more important to genuinely want to help than to be of the same age, gender or cultural/religious background as the person. However, if you think the person is uncomfortable interacting with you due to differences in age group, gender, language, caste, religion and/or ethnic and cultural backgrounds, you should ask the person if they would prefer to talk to someone more like themselves.

You should be aware of the stigma associated with suicide. Also, in some cultures, males may be less likely to express their emotions. When asked, they may not openly disclose previous suicide attempts and instead state, for example, that they had an "accidental overdose of medication or poison". Females from some cultural backgrounds may not be in a position to seek professional help on their own and therefore family members may need to be involved. All these can act as barriers to opening up and disclosing suicidal intentions. However, these barriers should not stop you from trying to talk to the person if you have concerns.

If the person is having trouble communicating with you, you should speak slowly, use simple words and, if necessary, repeat what you have said.

How should I talk with someone who is suicidal?

Tell the person that you care and want to help, and that you do not want them to die. It is more important to show you really care than to say 'all the right things'.

Be supportive and understanding of the person and listen to them with all your attention. Suicidal thoughts are often an appeal for help and a desperate attempt to escape from problems and distressing feelings. You should give the person a chance to talk about those feelings and their reasons for wanting to die. Explore the person's attitude towards suicide (e.g. suicide is a heroic thing to do). Discuss relationship issues, sexual interactions and related concerns, especially with females.

Ask the suicidal person what they are thinking and feeling. Tell them that you want to hear whatever they have to say. Let the person know it is okay to talk about things that might be painful, even if it is hard. However, be aware that the person may not be able to disclose some information with you, such as secrets that are unsafe to share. Reassure the person that it is not a crime, a sin or shame to feel suicidal. Recognise and be understanding and respectful of the suffering of the person. Give them a chance to express their thought and feelings (e.g. allow them to cry, express anger or scream), explain their reasons for wanting to die and acknowledge these (e.g. show you are listening). A person may feel better because they have told someone what they are thinking and feeling.

Remember to thank the suicidal person for sharing their feelings with you and talk about the courage it takes to do this.

See Box 2 Listening tips for tips on how to listen effectively and Box 3 What not to do

Box 2

Listening tips

- ***Be patient and remain calm and in control while the suicidal person is talking about their feelings. Encourage the person to do most of the talking.***
- ***Listen to the person without expressing judgment.***
- ***Find out more about the suicidal thoughts and feelings and the problems behind them by asking open questions that cannot be answered with a simple 'yes' or 'no'.***
- ***Keep in mind that asking too many questions can bring on anxiety (nervousness, fear) in the person. Show you are listening by repeating back to the person what they are saying.***
- ***Clarify important points with the person so that you know they understand.***
- ***Express empathy for the suicidal person.***

Box 3

What not to do

Don't

- argue or debate with the person about their thoughts of suicide
- dismiss the suicidal person's problems, or compare their problems to someone else's
- interrupt with stories of your own
- show you are not interested or show a negative attitude through your body language
- challenge the person to carry out their threats by daring them or telling them to 'just do it'
- try to diagnose a mental health problem
- try to take control and be directive, unless the person is at immediate risk.

Do not avoid using the word 'suicide'. It is important to discuss the issue directly without fear or expressing negative judgment. Speak about suicide using appropriate language (e.g. using the terms 'suicide' or 'die by suicide') and avoid using terms to describe suicide that promote negative attitudes, such as 'commit suicide' (meaning it is a crime or sin) or referring to past suicide attempts as having 'failed' or been 'unsuccessful' (meaning death would have been a positive outcome).

How can I tell how urgent the situation is?

Take all thoughts of suicide seriously and take action. Do not dismiss the person's thoughts as 'attention seeking' or a 'cry for help'. Determine the urgency of taking action based on identifying suicide warning signs, including the number and nature of warning signs, and major risk factors and reasons for suicide (e.g. recent stressful event, domestic violence, mental illness, alcohol misuse, substance abuse, previous suicide attempt or family history of suicide).

Determine whether someone has definite intentions to take their life, or whether they have been having more unclear suicidal thoughts, like "what's the point of going on".

To do this, ask the suicidal person about issues that affect their immediate safety:

- Whether they have a plan for suicide.
- How they intend to suicide, i.e. ask them direct questions about how, when and where they intend to suicide, including the specific means they intend to use. Beware that more lethal means (e.g. large quantity or type of pesticide) suggests a greater urgency of risk.
- Whether they have already taken steps to get what they need to end their life.
- Whether they have ever attempted or planned suicide in the past or, if necessary, ask their significant others (e.g. family members or religious leader).
- Whether there have been changes in their employment or schooling, social life or family.
- Ask the person about their concerns for any children they may have and try to ascertain if the children are included in their suicidal plan.

Ask the person if they have been using drugs or alcohol. Intoxication (getting drunk or high on drugs) can increase the risk of a person acting on suicidal thoughts.

If the suicidal person says they are hearing voices, ask what the voices are telling them. This is important in case the voices are relevant to their current suicidal thoughts.

Ask the person how they would like to be supported and if there is anything you can do to help. It is also useful to **find out what has supported the person in the past and what supports are available to them:**

- Have they told anyone about how they are feeling?
- Are there people they can turn to when they need help or support?
- Is there anything important in the person's life that may reduce the immediate risk of suicide (e.g. attachments to children)?
- Have they received help for emotional or mental health problems or are they taking any medication?

Remember that people at the highest risk for acting on thoughts of suicide in the near future are those who have a specific suicide plan (i.e. the means, a place, a time and an intention to do it). However, the lack of a plan for suicide is not a guarantee of safety.

How can I keep the person safe?

Once you have established that a suicide risk is present, you need to take action to keep the person safe. If you think there is an immediate risk of the person acting on suicidal thoughts, act quickly, even if you are unsure. Work together with the person to ensure they are safe, instead of acting alone to prevent suicide.

Suicidal people often believe they have no choice but to die by suicide. Remind the person that they have some control over their suicidal thoughts; these don't have to be acted on and they are usually temporary. Clearly state that thoughts of suicide may be associated with a treatable disorder, as this may instill a sense of hope.

Encourage the person to talk about their reasons for dying and their reasons for living. Acknowledge that they are considering both options and emphasise that living is a real option for them. Encourage the person to consider the consequences of suiciding. Tell them that they are loved and would be missed.

Ask about the problems the person is facing and how you can help, including any risk of harm from someone else. Reassure them that there are solutions to problems or ways of coping instead of suicide. By talking about specific problems, you can help the person to feel hope that there are ways of dealing with the difficulties that seem never ending. Ask about the problem, try to identify available resources, suggest solutions and help the person to make a plan of action.

When talking to the suicidal person, focus on the things that will keep them safe for now, rather than the things that put them at risk. Talk about the 'good things' in a person's life, their hopes for the future, and other reasons to live. Encourage the person to think about their personal strengths and qualities, and the positive things in their life.

Encourage the person to take part in an activity that they have found has helped them cope in the past or that they enjoy. Make a list with the person of other things they can do to distract themselves, especially things that are relatively easy to do and that will increase a sense of control and achievement. Also encourage the person to spend time with their significant others (e.g. family, friends or religious leaders).

Make sure that potentially harmful items are not available to the suicidal person .Remove access to them, after you gain their trust and if it is safe to do so. If the suicidal person refuses to hand over the things they intend to use to kill themselves, ask them to move the items to a safer location or try to remove the person from the risky environment.

With the person's agreement, you should inform the community mental health workers or psychosocial workers in the area about the situation. If the person accepts to keep in touch with you, you should agree about an appropriate communication method.

Work out a plan to help keep the suicidal person safe (see Box 4 **Safety plan**). Involve the person as much as possible in decisions about the plan. However, do not assume that a safety plan is enough to keep the suicidal person safe.

Although you can offer support, you are not responsible for the actions or behaviours of someone else, and cannot control what they might decide to do.

Box 4

Safety plan

A safety plan is an agreement between the suicidal person and the first aider that involves actions to keep the person safe. If the person agrees, you should involve someone the person trusts in developing the safety plan. This might be a friend, family member, mental health professional or a religious or spiritual leader. The plan should be developed taking into account the suicidal person's sociocultural background. Work with the person to create plans to ensure their safety for the next 24, 48 and 72 hours.

The safety plan should:

- ***Include an agreement that the suicidal person does not attempt suicide.***
- ***Focus as far as possible on what the person should do rather than what they should not.***
- ***Clearly outline what will be done, who will be doing it, and when it will be carried out.***
- ***Include a list of contact numbers that the person agrees to call if they are feeling suicidal (e.g., the person's doctor or mental health care professional, a suicide helpline or 24 hour crisis line, and friends and family members who will help in an emergency).***
- ***The contact numbers should be kept somewhere accessible to the person.***
- ***If the person won't make a safety plan, it is not safe to leave them alone for any period of time and you should make sure someone stays close by the person (in the same room) and get outside help immediately. If necessary, get professional help.***

If there is concern about danger to the suicidal person's children, the safety plan should include contact details for relevant emergency services.

What about professional and other help?

Reassure the person by letting them know that we all go through tough times and need support and that reaching out for help is the first step to feeling better.

Ask the person if they would like for you to contact someone for them such as a friend, family member, or trusted religious, spiritual or community leader.

Encourage the person to get suitable professional help as soon as possible. Don't assume that the person will get better without help or that they will seek help on their own. Find out about the resources and services available to help a person who is considering suicide, including hospitals, mental health clinics, mobile outreach crisis teams, suicide prevention helplines and local emergency services.

Provide this information to the suicidal person and discuss help-seeking options with them.

Ask the person for permission to contact their regular doctor or, if applicable, mental health professional about your concerns. If possible, the health professional contacted should be someone the suicidal person already knows and trusts. If the person does not want to talk to someone face-to-face, encourage them to call a suicide helpline.

What if the suicidal person is reluctant or refuses to seek help?

You should be patient and persistent in encouraging them to get help.

Make sure someone who is close to the suicidal person is aware of the situation (i.e. a close friend or family member) and knows the relevant warning signs and how to assist the person. Contact a suicide prevention hotline or talk to a health professional for advice on how to help them.

It is particularly important to ensure that an adolescent receives help from a health professional, support group or a relevant community organization. Seek an agreement with the adolescent that they will contact a specific person within a specified timeframe. If you are unable to persuade them to get help, you should get assistance from someone they trust, such as a helpline or a mental health professional.

Be prepared that the person may get angry and feel betrayed by your attempt to prevent their suicide or help them get professional help, but try not to take personally any hurtful actions or words of the person.

What if the suicidal person has a weapon?

You should ask them to hand over the weapon, but make sure you do not put yourself in any danger.

What if the person wants me to promise not to tell anyone else?

Try to convince the person that it is better to not keep their suicidal intentions a secret but to talk to someone else (e.g. a professional or a family member). If the person is a minor, you must make their guardians (i.e. the family or significant elders) aware of the person's intention to kill themselves. Do not try take on the minor's responsibilities yourself.

Treat the person with respect and involve them in decisions about who else knows about the suicidal crisis and which details should be kept confidential. If you decide to involve a professional or someone else, let the person know you are doing this and explain that it is necessary to ensure their safety or someone else's.

What should I do if the person has acted on suicidal thoughts?

If the suicidal person has already harmed themselves, give them first aid, call emergency services and ask for an ambulance.

Remember, despite our best efforts, it is not always possible to prevent suicide.

Self-injury for reasons other than suicide

Never assume that a person who self-harms is suicidal, as some people injure themselves for reasons other than suicide. If you are unsure whether injuries are due to a suicide attempt, you should ask the person directly.

For some people, self-injury is intended to relieve unbearable anguish, to stop feeling numb or other emotional reasons. This can be distressing to see. There are *First aid guidelines for non-suicidal self-injury* (http://mhfa.com.au/file/1142/download) which, although not developed specifically for people in Sri Lanka, can help you to understand and assist if this is occurring.

Take care of yourself

After helping someone who is suicidal, make sure you take appropriate self-care. Providing support and assistance to a suicidal person is exhausting and it is therefore important to take care of yourself.

AN IMPORTANT NOTE

Purpose of these Guidelines

These guidelines are designed to help members of the public to provide first aid to someone in Sri Lanka who is at risk of suicide. The role of the first aider is to assist the person until appropriate professional help is received or the crisis resolves.

Development of the Guidelines

The guidelines are based on the expert opinions of a panel of experts with professional experience with mental health and suicide prevention in Sri Lanka about how to help someone who may be at risk of suicide. The methodology was based on Ross AM, Jorm AF, Kelly CM. Re-development of mental health first aid guidelines for suicidal ideation and behaviour: A Delphi study (https://mhfa.com.au/cms/mental-health-first-aid-guidelines-project).

How to use these Guidelines

These guidelines are a general set of recommendations about how you can help someone in Sri Lanka who may be at risk of suicide. Each individual is unique and it is important to tailor your support to that person's needs. These recommendations therefore may not be appropriate for every person who may be at risk of suicide.

More resources about how to discuss suicide are available at www.conversationsmatter.com.au Although not developed specifically for people in Sri Lanka first aiders may find them useful.

These guidelines were produced in collaboration with the National Institute for Mental Health, Sri Lanka.

Although these guidelines are copyright, they can be freely reproduced, made available online or electronically, for non-profit purposes provided the source is acknowledged. Please cite the guidelines as follows:

Suicide First Aid Guidelines for Sri Lanka (2014) Melbourne: Global and Cultural Mental Health Unit, Centre for Mental Health, The Melbourne School of Population and Global Health, The University of Melbourne.

Enquiries should be sent to: Dr Erminia Colucci ecolucci@unimelb.edu.au, Global and Cultural Mental Health Unit (GCMHU), Centre for Mental Health, The University of Melbourne.

These guidelines can be downloaded from GCMHU website (http://mspgh.unimelb.edu.au/centres-and-institutes/centre-for-mental-health)**.**

All MHFA guidelines can be downloaded from www.mhfa.com.au

THE UNIVERSITY OF MELBOURNE

MELBOURNE SCHOOL OF POPULATION & GLOBAL HEALTH

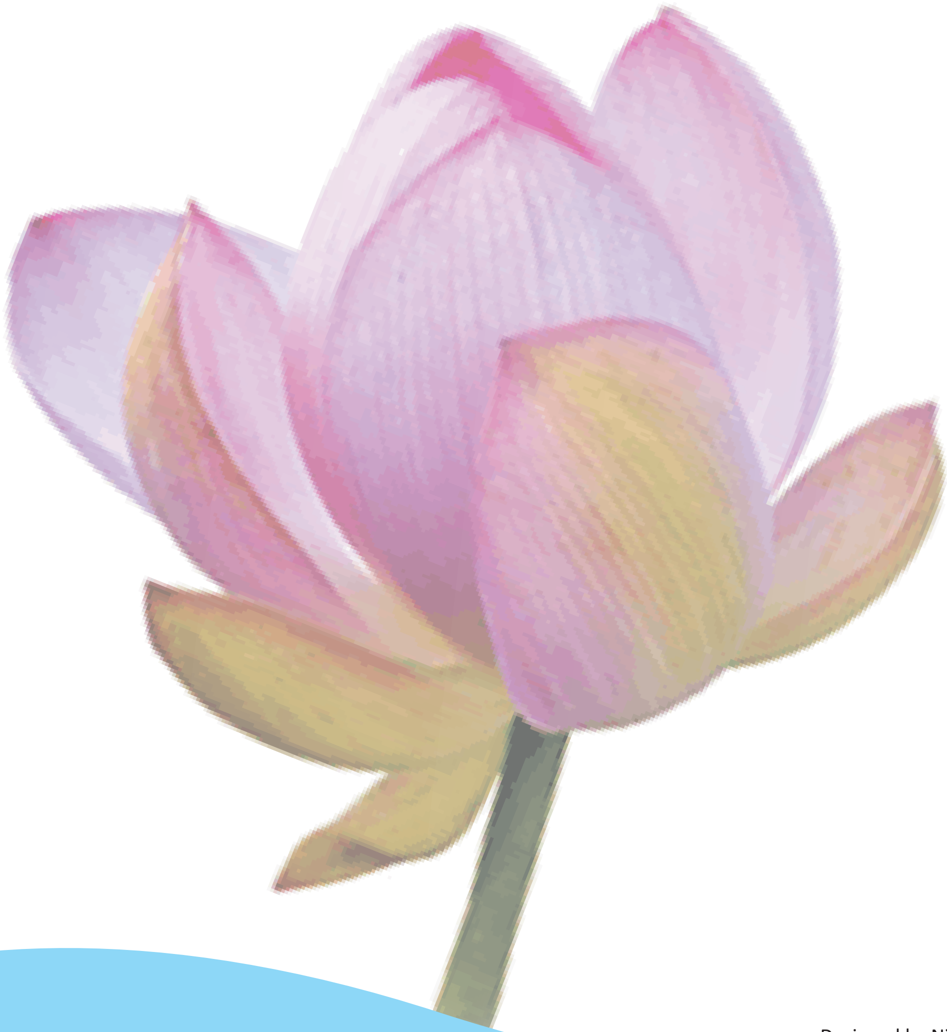

Designed by Niranka Madushan
